# The COVID-19 pandemic: doom to international medical electives? Results from two German elective databases

**DOI:** 10.1186/s13104-021-05708-3

**Published:** 2021-07-26

**Authors:** Abdullah Egiz, Maximilian Andreas Storz

**Affiliations:** 1 School of Medicine, University of Central Lancashire, Preston, UK; 2 Center for Complementary Medicine, Faculty of Medicine, University of Freiburg, Freiburg im Breisgau, Germany

**Keywords:** COVID-19 pandemic, SARS-CoV-2, Elective, Medical Student, Clerkship, International Health, Global Health, Public Health

## Abstract

**Objective:**

International medical electives are an essential part of medical education and popular among medical students. During the COVID-19 pandemic, however, many students had assistantship placements postponed and electives cancelled. Educational institutions switched face-to-face campus-based teaching to virtual platforms. Although it is conceivable that international medical electives were particularly affected by this development, numerical data on this phenomenon is yet scarce. To investigate how the COVID-19 pandemic influenced the clinical elective behavior of German-speaking medical students, we systematically analyzed two large German online databases (Famulatur-Ranking and PJ-Ranking) cataloging medical elective experience testimonies.

**Results:**

The COVID-19 pandemic substantially reduced the number of German medical students undertaking abroad medical electives. Between 2018 and 2020, a total of 10,976 reports were uploaded to both databases. We observed a notable decline in abroad elective reports in 2020. Prior to the COVID-19 pandemic, almost 5% of reports uploaded to “PJ-ranking” covered an international medical elective. This number dropped to 1.68% in 2020. Analyzing “Famulaturranking”, we observed a comparable phenomenon. While 4.74% of reports in 2019 covered an international elective, the number dropped to 2.02% in 2020. The long-term consequences of this phenomenon will be subject to future research.

## Introduction

The COVID-19 pandemic has caused an unprecedented interference in medical education [1]. During the last months, many countries took uncompromising measures in response to the SARS-CoV-2 outbreak. These measures included the introduction of social and physical distancing, banning of public events [2] the prevention of non-essential travel and ultimately “national lockdowns” [1]^2^. Schools and universities had to close which made it impossible for educational institutions to continue lectures as usual [3]. This situation prompted medical educators to respond at local and national levels [4] and educational institutions often switched face-to-face campus-based teaching to virtual platforms [3–5].

The rapid change in the medical education process is challenging for medical educational bodies and students. Medical schools and teaching hospitals must deliver teaching safely [3] whilst also ensuring the reliability and continuity of clinical education [1]^2^ This might, above all, affect clinical rotations where interpersonal contact is inevitable [6].

A British survey reported that many students had assistantship placements postponed and electives cancelled [2]. Lucey and Johnston argued that quarantine restrictions and institutional challenges in identifying sufficient clinical training sites for their students forced medical schools to suspend their usual practice of offering visiting elective rotations for senior students [4].

It is conceivable that the COVID-19 pandemic may therefore lead to a substantial reduction in abroad medical electives. However, data underpinning this assertion is yet scarce. Thus, we analyzed two German elective reports databases to investigate how the COVID-19 pandemic affected the international abroad elective frequency in students from the German-speaking countries.

## Main text

### Materials and methods

To undermine our hypothesis, we used two popular German online databases for medical elective experience testimonies. The free online database “Famulaturranking” (www.famulaturranking.de) allows students to rate their medical electives and to share their experience by uploading elective reports. In Germany, medical students are required to complete 4 one-month elective clerkships during the clinical phase of their studies [7] 8. These clerkships are traditionally called “Famulatur”, originating from the Latin word “famulus”, which translates as “servant” [7].

It is popular among German students to perform at least one elective outside Germany. Upon completion, students frequently rate their experience at a particular hospital via “Famulaturranking”. The categories “country”, “city”, “hospital” and “duration” are mandatory when a student uploads his rating. All reports are anonymous. The database is in German language and is mainly used by German, Austrian and Swiss students. For our analysis, we defined an abroad elective as an elective that was performed outside of Germany, Switzerland and Austria.

In a second step, we examined another German online database which is specifically designated for final year medical elective experience reports. This database is called “PJ Ranking” (www.pjranking.de). “PJ” is the German abbreviation for “Praktisches Jahr”, which can be translated as “practical year”. This final stage of medical school is divided into three full-time clinical rotations, each lasting approximately 4 months [7] 8. While rotations in Surgery and Internal Medicine are mandatory, one rotation can be freely chosen from all the clinical specialties [7]. The specific time frames for each rotation are determined individually by the German universities.

We analyzed the years 2018, 2019 and 2020. Data extrapolation was performed in the last week of December 2020. Reports were copied to a Microsoft Excel file and analyzed using statistical software PSPP (GNU PSPP [Version 0.8.5]. Free Software Foundation, Boston). Duplicates of identical reports that were uploaded twice or more were removed.

### Results

**Fig. 1:**
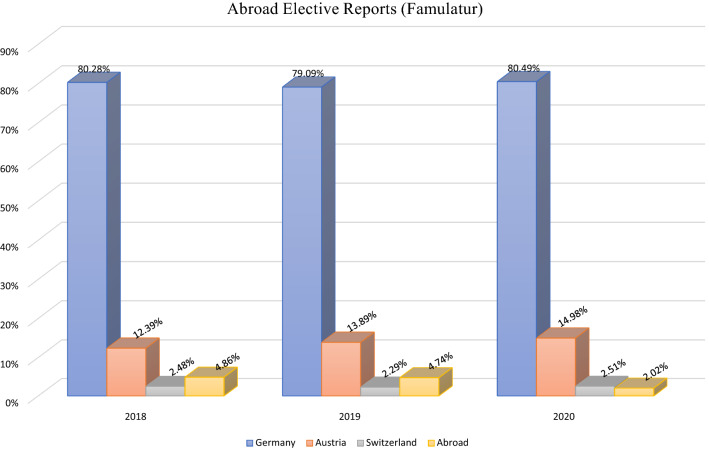
Distribution of clerkship reports (Famulatur) between 2018 and 2020, obtained from www.famulaturranking.de

3425 reports were uploaded to “Famulaturranking” between 2018 and 2020. 1009 reports were uploaded in 2018, 1181 reports in 2019 and 1235 reports in 2020 respectively. Figure 1 shows that the number of students reporting an elective in a non-German speaking country substantially declined during the COVID-19 pandemic (January 2020 till present).

While 56 (56/1181) abroad elective experience reports were uploaded in 2019, the number fell to 25 (25/1235) in 2020. Conversely, the number of students reporting an elective in their home country increased (Fig. 1).

**Fig. 2:**
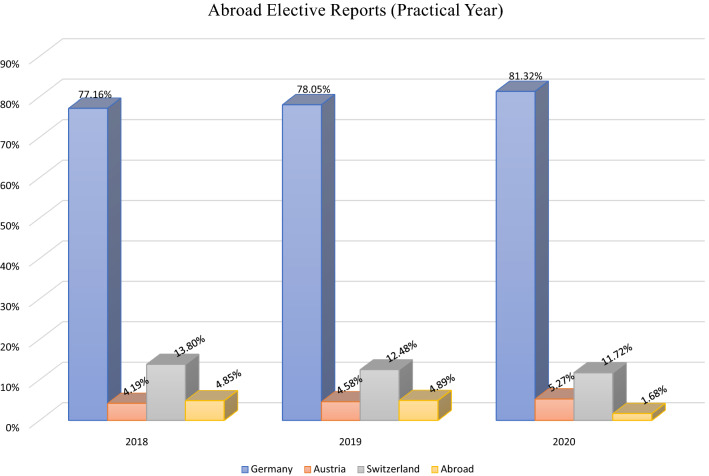
Distribution of final year elective reports (Practical Year) between 2018 and 2020, obtained from www.pj-ranking.de

Analyzing the elective database “PJ Ranking”, we identified another 7551 elective reports that were uploaded between 2018 and 2020. Again, we observed a notable decline in abroad elective reports in 2020 (Fig. 2). Only 1.68% (30/1783) of testimonies reported an international medical elective. Prior to 2020, almost 5% of reports (156/3189) covered an abroad medical elective. This decline was more pronounced in final year electives compared to “Famulatur” clerkships.

### Discussion

International medical electives have been associated with a large number of perceived benefits, including improved clinical examination skills, less dependency on expensive technology, a better understanding of tropical diseases and enhanced cross-cultural understanding [9]^10^,^11^. Using two German databases, we observed a substantial decline in abroad elective reports in 2020 in the German-speaking countries. Following the COVID-19 pandemic, the number of reported abroad electives dropped by more than 50% compared to 2019.

To the best of our knowledge, we are the first group to report such a decline in abroad elective reports in German-speaking medical students during the COVID-19 pandemic. Our results confirm an expected trend that has also been underlined by a recent report by Choi et al. [2], who emphasized that many students in the United Kingdom had electives cancelled during the pandemic.

Several explanations are conceivable for this phenomenon. Like most other countries, Germany, Austria and Switzerland have been heavily affected by travel restrictions. It is likely that many students had to cancel electives because they were unable to travel to their destination. Moreover, many institutions closed their international elective programs for foreign students. New policies were implemented to reduce unnecessary contacts and to prevent the spread of the virus. Therefore, students most likely encountered substantial difficulties to secure an abroad elective placement.

It is also likely that students now experience a lack of funding and have insufficient financial resources to go abroad. While international electives are usually expensive, many students lost their revenue streams during the pandemic. Bars, restaurants and other small businesses had to close during the national lockdowns. Thus, it is conceivable that many students lost their jobs and encountered difficulties to cover the fees associated with an elective.

Our findings might adversely affect students in various ways. The COVID-19 pandemic is a global health crisis and highlights the importance for future doctors to think globally. The lack of abroad elective opportunities could impede this and may hinder students’ career progression. Moreover, the current paucity of international electives may also interfere with professional identity formation [12].

International medical graduates who are candidates for a job post or interested in an international career are negatively affected as well [13]. Elective periods are usually the only way for students to gain clinical experience and represent a crucial opportunity to receive letters of recommendation at the country of interest. Many residency programs require a minimum length of country-based hands-on clinical experience and specify a certain number of mandatory references. Applying to these programs will certainly be harder for international medical graduates in the future. So-called “tele-rotations” were recently suggested as a potential solution to this, as they allow trainees to participate (in international electives) remotely from across the world and to connect with international health systems without travelling [14]. Whether tele-rotations may compensate for the lack of in-person electives is currently subject to a controversial debate and additional research is necessary to evaluate its role in undergraduate education.

### Conclusion

The COVID-19 pandemic substantially reduced the number of German medical students undertaking abroad medical electives. The number of reported international electives in 2020 dropped by more than 50% compared to 2019. The long-term consequences of this phenomenon will be subject to future research.

## Limitations

Our analysis has strengths and limitations that warrant further investigation. The large dataset behind this submission is somewhat unique and to the best of our knowledge, there are no other international databases that include a comparably high amount of elective reports. On the other hand, only a fraction of German-speaking students regularly uploads elective reports. It is neither mandatory nor are students being given credit for it. Furthermore, the number of “PJ” reports for 2020 was lower compared to 2019, since the year was not completed when we extrapolated the data. Some rotations end in December and it is likely that some students had not yet uploaded their reports at this time. Nevertheless, glancing at the current (global) travel restrictions, we believe that the number of international electives did not change much during the last two weeks of December.

## Authors' information

Abdullah Egiz is an international medical student in the UK and interested in medical education. Maximilian Storz is a medical doctor from Germany and author of the book “PJ und Famulatur im Ausland”.

## Data Availability

Data were obtained from Famulaturranking (www.famulatur-ranking.de) and PJ-ranking (www.pj-ranking.de). Public access to both databases is open. Please contact the corresponding author (MAS) for any data requests and other related inquiries. All data associated with this paper will be made available upon reasonable request.
